# New Insight for the Genetic Evaluation of Resistance to Ostreid Herpesvirus Infection, a Worldwide Disease, in *Crassostrea gigas*

**DOI:** 10.1371/journal.pone.0127917

**Published:** 2015-06-03

**Authors:** Lionel Dégremont, Jean-Baptiste Lamy, Jean-François Pépin, Marie-Agnès Travers, Tristan Renault

**Affiliations:** 1 Institut Français de Recherche pour l’Exploitation de la Mer, Laboratoire de Génétique et de Pathologie des Mollusques Marins, Avenue Mus de Loup, La Tremblade, France; 2 Institut Français de Recherche pour l’Exploitation de la Mer, Laboratoire Environnement Ressources des Pertuis Charentais, Avenue Mus de Loup, La Tremblade, France; Bigelow Laboratory for Ocean Sciences, UNITED STATES

## Abstract

The Pacific oyster, *Crassostrea gigas*, is the most important commercial oyster species cultivated in the world. Meanwhile, the ostreid herpesvirus 1 (OsHV-1) is one of the major pathogens affecting the Pacific oyster, and numerous mortality outbreaks related to this pathogen are now reported worldwide. To assess the genetic basis of resistance to OsHV-1 infection in spat *C*. *gigas* and to facilitate breeding programs for such a trait, if any exist, we compared the mortality of half- and full-sib families using three field methods and a controlled challenge by OsHV-1 in the laboratory. In the field, three methods were tested: (A) one family per bag; (B) one family per small soft mesh bag and all families inside one bag; (C) same as the previous methods but the oysters were individually labelled and then mixed. The mean mortality ranged from 80 to 82% and was related to OsHV-1 based on viral DNA detection. The narrow-sense heritability for mortality, and thus OsHV-1 resistance, ranged from 0.49 to 0.60. The high positive genetic correlations across the field methods suggested no genotype by environment interaction. Ideally, selective breeding could use method B, which is less time- and space-consuming. The narrow sense heritability for mortality under OsHV-1 challenge was 0.61, and genetic correlation between the field and the laboratory was ranged from 0.68 to 0.75, suggesting a weak genotype by environment interaction. Thus, most of families showing the highest survival performed well in field and laboratory conditions, and a similar trend was also observed for families with the lowest survival. In conclusion, this is the first study demonstrating a large additive genetic variation for resistance to OsHV-1 infection in *C*. *gigas*, regardless of the methods used, which should help in selective breeding to improve resistance to viral infection in *C*. *gigas*.

## Introduction

The Pacific cupped oyster *Crassostrea gigas* is cultivated in numerous countries around the world, and its production was estimated to be 0.6 million tons in 2012, without taking into account an estimated 4 million tons from Chinese oyster production, some of which was *C*. *gigas* [1]. This species has been subjected to mortality outbreaks related to various diseases, and ostreid herpesvirus 1 (OsHV-1) is one of the major pathogens affecting the Pacific oyster. During the last decade, numerous reports of mortality outbreaks related to OsHV-1 in oysters have been reported worldwide, from the west coast of the USA [2] to New Zealand and Australia [3–7], as well as in Europe, including Ireland [8], Spain [9], Portugal [10] and Italy [11]. However, the most impacted country is France, the fourth highest world producer of *C*. *gigas*, where OsHV-1 has regularly affected cultivated spat and juveniles for many years, with dramatic mortality outbreaks reported each year since 2008 [12–14]. It is also suspected that OsHV-1 was one of the major causes of the mortality events reported in spat and juvenile *C*. *gigas* during the Morest program in France, which aimed to better understand the summer mortality phenomenon affecting *C*. *gigas* through a multidisciplinary approach [15]. During this program, OsHV-1 was detected in moribund spat sampled during mortality outbreaks in field, nursery and laboratory trials in 2001, 2002 and 2003. This hypothesis was recently reinforced by the comparison of mortality in 2001–2003 with those observed since 2008 using the same oyster lines, which showed a higher susceptibility of spat than of adults [12, 16] by a similar susceptibility between diploids and triploids [17, 18] and, in particular, by the evidence of a higher OsHV-1 infection resistance or susceptibility in oysters selected for their higher or lower survival to the summer mortality phenomenon, respectively [19, 20].

During the Morest program, the heritability of survival was estimated for spat *C*. *gigas* in field conditions using sib-analysis from a hierarchical mating design, as well as through a divergent selection [21, 22]. It was also observed through high positive correlations between survival in the field and survival in the laboratory during mortality outbreaks, with the detection of OsHV-1 DNA in the laboratory trials [17].

The genetic evaluation of the oyster families from 2001 to 2003 was performed using numerous bags in the field, which was space- and time-consuming. Basically, one oyster family is placed into several bags, and such evaluations are known to increase the environmental component of variation among families [23]. Thus, as an alternative to the previous genetic evaluation, all oyster families could be placed into one bag, which could be then replicated to mimic a communal stocking evaluation, as has been developed for fish [24]. Such a design minimizes the covariance between genetic and environmental effects, increasing the accuracy of predicted breeding values [25]. Nevertheless, this method is valid only when certain genetic, environmental and physiological criteria are met (in our case, under OsHV-1 infection pressure) and when the ranking of the families between the communal and separate evaluations is the same [23].

Laboratory trials, developed in 2001 and then run in 2002 and 2003, were performed to test resistance to the summer mortality phenomenon of several or all families in only one tank, but without estimating the genetics parameters [17]. As indicated above, OsHV-1 DNA was detected for the first time in oysters tested in the laboratory trials without experimental infection, and high positive correlations were observed between mortality in the field and mortality in the laboratory [17]. Thus, the genetic evaluation of resistance to OsHV-1 infection in oyster families can be obtained through an experimental infection in laboratory trials. Such methods have been developed and are well described [26, 27]. The last step is to compare the genetic evaluation under field and laboratory conditions to validate both methods for evaluating the resistance to viral infection in oyster families.

We report in this study three testing methods to evaluate the capability of survival in oyster families in field conditions during a mortality outbreak related to OsHV-1 DNA detection. Additionally, families were experimentally infected in controlled conditions in the laboratory. From field and laboratory evaluations, testing methods were compared, and heritability, as well as genetic correlations, were calculated for mortality related to OsHV-1 infection.

## Materials and Methods

### Biological materials

Wild oysters were sampled on an oyster bar located in La Tremblade in December 2010 (45°46'53.5" N, 1°7'19.5" W), which were then brought to the Ifremer hatchery in La Tremblade. These broodstock oysters were placed in a 240-L conditioning raceway with a flow of 400-L·h^-1^. The seawater temperature was gradually increased from ambient temperatures to 21°C for one week, and the oysters were then held at this temperature until spawn. The seawater was enriched with a cultured phytoplankton diet (*Isochrysis galbana*, *Tetraselmis suecica* and *Skeletonema costatum*) to favor gametogenesis.

The spawn occurred on March 14^th^, 2011. The oysters were opened, and those not ripe were discarded. The sex was determined using a microscope and by taking a small sample from the gonad and spreading it on a slide. For each parent, the gametes were collected by stripping the gonad, which were successively sieved to remove large (>60 μm) and small (<20 μm) tissue debris for the eggs and to only remove the large (>60 μm) ones for the sperm. A total of 24 males were each mated to 2 females, the 24 males and 48 females were randomly sampled and randomly mated, producing 24 half-sib families (HSF) each containing 2 full-sib families (FSF). In addition, a selected FSF “R”, using selected broodstock for its higher resistance to summer mortality, which was also characterized for its higher resistance to OsHV-1 infection in field conditions, was also produced as a control for the FSF [20]. Each family was reared in a 30-L tank at 25°C in filtered and UV-treated seawater, which was changed 3 times per week. The larvae were fed daily with *I*. *galbana*, and *S*. *costatum* was also added when the larvae size was 140 μm. The larval stage was successful for 46 FSF and the FSF “R”, and settlement occurred 14 to 20 days post-fertilization. Competent larvae were settled on cultch in 120-L raceways and reared under standard hatchery conditions, and when spat reached 2 mm, they were transferred to the Ifremer nursery in Bouin (France) on May 2^nd^, 2011. The oysters were then grown in unheated seawater enriched with *S*. *costatum*. The families remained in the nursery until their evaluation in the field and in the laboratory.

### Field study

At deployment, the oysters were 4.5 months old, and the mean average of the individual weights was 2 g. Thirty oysters were randomly sampled among the FSF for the detection of OsHV-1 DNA. All families (22 HSF containing each 2 FSF, and 2 FSF for which the corresponding half-sib families failed at the larval stage) were deployed on August 1^st^, 2011, in our oyster farm at Agnas in the Marennes-Oléron Bay (45°52’23”N, 1°10’15”W), where numerous oyster farms surround our experimental farm, and the experiment was terminated on September 1^st^, 2011. The oyster farm used is our study is the property of Ifremer and no specific permissions were required. The oysters were tested using bags attached to racks, which emerged when the tide coefficient was above 70 (the difference in height between the low and high tide was then 3.65 m). The seawater temperature was recorded every hour throughout the study using two ThermoTrack probes (Progesplus, 59780, Willems, France).

Three testing methods were used to evaluate the survival of the families, but unfortunately, all families could not be tested with the three methods, as a result of the lower fecundity of the parents of some families. The first method is commonly used for the evaluation of our oyster lines. Forty-two FSF were tested, and each family was represented by 2 bags of 150 oysters each, representing a total of 84 bags, which were randomly placed on the racks (Table 1); the FSF “R” was also tested according to this method. The second method consisted of putting 50 oysters per family inside of a soft mesh bag, and all of the families were then placed into one bag. All 46 FSF were evaluated according to this method, corresponding to 2300 spat inside one bag. This was replicated three times (Table 1). The last method of evaluation was similar to the previous one, except that oysters were individually labelled with the family number on the shell using either a permanent marker or a tag attached with epoxy resin; thus, 41 FSF were mixed within the bag. This was repeated three times (Table 1). The three field testing methods were named BF, MF and LF for bag, mesh and label, respectively.

**Table 1:** Summary of the four testing methods to evaluate the mortality and OsHV-1 resistance for *Crassostrea gigas* spat.

Site	Method of evaluation^a^	Number of full-sib families	Number of families per replicate	Number of replicates	Number of oyster per family and per replicate	Number total of replicates	Number total of oysters evaluated
Field	BF	42	1	2	150	84	12 600
Field	MF	46	46	3	50	3	6 900
Field	LF	41	41	3	50	3	6 150
Laboratory	OsHV-1 challenge^b^	44	1	4	10	176	1 760

^a^ BF indicates that one family was in one bag; MF indicates that all of the families were in one bag, but the families were separated inside with a mesh; LF indicates that all of the families were in one bag and the oysters were individually labeled and then mixed.

^b^ OsHV-1 challenge was performed through injection for each of the 1760 oysters tested

Two weeks post-deployment, 30 moribund oysters for each testing method were randomly sampled among several FSF for the detection of OsHV-1 DNA. Finally, survival was recorded for all oysters by counting the dead and live oysters one month post-deployment. Unfortunately, a clear identification of the family number was not possible for 35% of the oysters tested by the LF method due to the erasure of the marker, and only those with a clear identification were kept for the estimation of survival.

### Experimental infection with OsHV-1 under controlled conditions

The susceptibility of 44 FSF to OsHV-1 infections was tested through experimental infection under controlled conditions in October-November 2011 when the oysters were 7 months old. The average mean individual weight was approximately 5 g. The intramuscular route was selected for infectious challenge using a protocol already described [26, 27]. For each family, 40 oysters were anaesthetized for 4 h in a solution containing magnesium chloride (MgCl_2_, 50 g/L) in seawater (1 v)/ tap water (4 v) [28]. One hundred microliters of an OsHV-1 suspension (OsHV-1 μVar genotype, GenBank HQ842610-1) at 6 x 10^3^ copies of viral DNA/μL were injected into the adductor muscle. Each family was then placed in four tanks (10 oysters per tank) containing 5 L of UV-treated and filtered seawater (1 μm) at 22°C with gentle aeration, without a food supply and without flow-through for 5 days. The temperature of the room was kept constant at approximately 22°C for the duration of the experiment. A group of control animals, randomly sampled among six FSF, was used (i) as a positive control when injected with viral suspension, 10 tanks with 10 animals each, or (ii) as a negative control when injected with filtered and sterile seawater, with four tanks with 10 animals each. Cumulative mortalities were recorded by counting the dead and live oysters at day 5 post-injection.

### OsHV-1 DNA detection and quantification

The detection and quantification of OsHV-1 DNA was carried out using the SYBR green real-time PCR protocol described in [29], which was adapted with DPFor/DPRev primers to target the OsHV-1 DNA polymerase sequence (ORF 100) [30]. The results were expressed as the viral DNA copy number per mg of oyster tissue. The detection and quantification of OsHV-1 DNA was performed for each of the individuals sampled at the nursery (live oysters) and in the field (moribund oysters).

### Statistical analyses

The studied trait was binary (dead = 0 and live = 1) for both field monitoring and experimental infection, and analyses were based on mortality at the endpoint for each method. The genetic analyses were performed with a generalized linear mixed model (GLMM) using the SAS Glimmix procedure with a logit link using the Laplace method to approximate the log likelihood [31]. The data used and the SAS code are provided in S1 Table and S1 Text respectively.

The model for the whole dataset (i.e., the four methods [BF, LF MF and OsHV-1 challenge]) are defined below:

$$\text{Logit}\;\left(Y_{ijkl}\right)=\mu_{i}\;+\;\mu_{j}\;+\;\mu_{k(j)}\;+\;\mu_{i}\;\text{x}\;\mu_{j}\;+\;\mu_{i}\;\text{x}\;\mu_{k(j)}$$

where *Y*_*ijkl*_ = observed mortality at the endpoint for the oyster *l* in the family, bred using the female *k* (1 to 48) nested within the male *j* (1 to 24) in method *i* (BF, LF MF and OsHV-1 challenge),

*μ*_*i*_ = fixed effects of the testing method,

*μ*_*j*_ = random effects of males,

*μ*_*k(j)*_ *=* random effects of female nested within the male

*μ*_*i*_ x *μ*_*j*_ *=* random interactions between males and methods

*μ*_*i*_ x *μ*_*k(j)*_ *=* random interactions between females nested within the male and methods

The models used for each method were:

For BF and OsHV-1challenge: Logit (*Y*_*jkln*_) = *μ*_*j*_ + *μ*_*k*(*j*)_ + *μ*_*n*(*kj*)_

For MF and LF: Logit (*Y*_*jkln*_) = *μ*_*j*_ + *μ*_*k*(*j*)_ + *μ*_*n*_

where the parameters are defined above and *μ*_*n(kj)*_ *=* random effects of replicate nested within female and male (n = 2 for BF, and n = 4 for OsHV-1 challenge) and *μ*_*n*_ *=* fixed effects of the replicate (n = 3).

Genetic parameters were computed directly from the GLMM estimators as they were given in the underlying liability scale. Narrow sense heritability, broad heritability and the combined estimation of heritability were calculated, respectively, as follows [32]:

- h^2^n=Va /Vt = 4σ²m / (σ²m + σ²f + σ²e)

where Va is the additive genetic variance, estimated as four times the variance among males (σ²m), and Vt is the total phenotypic variance, with σ²e being π²/3 for a logit link.

- h^2^b = Vg/Vt = 4σ²f / (σ²m + σ²f + σ²e)

where Vg is the genetic variance, estimated as four times the variance among females (σ²f), which include additive, dominance and common environment components.

- h² m+f = 2×(σ²m + σ²f) / Vt

The narrow and broad genetic correlations for mortality between methods were calculated from the half-sib and full-sib family means (logit transformed) to compute genetic covariances between methods while genetic variances were those calculated from the GLMM procedure with the logit transformation.

Additionally, heritabilities for mortality were calculated with a mixed model using the SAS Mixed procedure for binary data. Variance components and heritability were obtained on the observed scale, and the latter was then converted to the underlying scale [33]. The SAS code is given in S1 Text and the results are presented in S2 Table. Genetic correlations for mortality between methods were also calculated from the estimates of breeding values (EBV) using best linear unbiased prediction (BLUP) analysis and the results are presented in S3 Table.

## Results

### Field study

The seawater temperature during August 2011 was 21.1 ± 0.8°C. At deployment, OsHV-1 DNA was not detected in any of the 30 oysters screened. Two weeks post-deployment, mortality was observed in all three testing conditions, and OsHV-1 DNA was detected in 27, 30 and 29 moribund oysters screened from the BF, MF and LF methods, respectively, with corresponding viral loads of 2.2 10^+6^, 2.0 10^+6^ and 5.3 10^+6^ copies of OsHV-1 DNA per mg of oyster tissue (Table 2).

**Table 2:** Mean mortality (±SD), mortality range among the full-sib families and OsHV-1 results

				OsHV-1	
Site	Method of evaluation^a^	Mortality (%)	Mortality range (%)	Detected positive/total analyzed	Mean viral load (DNA copy per mg of oyster tissue)
Nursery	_	_	_	0/30	_
Field	BF	81.7 ± 18.5	31–100	27/30	2.2 10^+6^
Field	MF	80.4 ± 17.8	30–99	30/30	2.0 10^+6^
Field	LF	80.2 ± 17.0	29–99	29/30	5.3 10^+6^
Laboratory	OsHV-1 challenge	57.6 ± 25.2	8–100	_	_

^a^ BF indicates that one family was in one bag; MF indicates that all of the families were in one bag but the families were separated inside with a mesh; LF indicates that all of the families were in one bag and the oysters were individually labeled and then mixed.

At the end of the experiment, the mean mortality among families was 82, 81 and 80% for the BF, MF and LF methods, respectively (Table 2). The mortality ranged from 29 to 100% among the FSF (Table 2) and was the lowest (12%) for the FSF “R”.

### Experimental infection with OsHV-1 under controlled conditions

Five days post-injection, the mean mortality among the FSF injected with the OsHV-1 suspension was 58%. The controls showed 58% mortality when injected with the OsHV-1 suspension, while no mortality was recorded for those injected with seawater only. Some families demonstrated low mortality rates (8%), whereas some succumbed totally (100%) (Table 2).

### Comparison of spat mortality in field and laboratory trials and genetic parameters for resistance to OsHV-1 infection

The narrow sense heritability estimated from the generalized linear mixed model was moderate for the four testing methods, ranging from 0.49 to 0.61, and was only significantly different from 0 for OsHV-1 challenge (*p*<0.05) (Table 3). Broad sense heritability was higher, with values ranging from 0.68 to 1.05 within the method, which were all significantly different from 0 (*p*<0.05). For the overall analysis, the narrow and broad sense heritability was 0.65 and 0.89 respectively (Table 3). Heritability estimated from sire and dam components had intermediate values (from 0.62 to 0.83) and lower standard error (Table 3). Similar trends were also found using the Dempster & Lerner method [33] (S2 Table). Genetic correlations between the testing methods used for field evaluation were all positive and high, ranging from 0.92 to 0.99 (Table 4). The genetic correlations between one of the field testing methods and the laboratory challenge were still positive and high, but to a lesser extent, ranging from 0.68 to 0.75 (Table 4). Similar trends were also found using the estimated breeding values from BLUP (S3 Table). The S1 Fig showed the narrow genetic correlation for mortality between methods.

**Table 3:** Variance components and narrow, broad-sense and combined estimation of heritabilities (h^2^ n, h^2^ b and h^2^ s+d, respectively) (S.E.) for mortality in *C*. *gigas* spat for each testing method in the field and in the OsHV-1 challenge in the laboratory.

Variance	BF	MF	LF	OsHV-1 challenge	Overall
Va	3.38±(2.52)	2.87±(1.67)	2.35±(1.54)	2.98±(1.66)	3.54±(2.07)
Vg	5.91±(2.21)	4.15±(1.40)	3.58±(1.35)	3.32±(1.33)	4.80±(1.50)
Verror^a^	3.29±(3.30)	3.29±(3.30)	3.29±(3.30)	3.29±(3.30)	3.29±(3.30)
Vphenotypic	5.61±(0.57)	5.05±(0.43)	4.77±(0.38)	4.86±(0.43)	5.38±(0.49)
h^2^ n	0.60±(0.42)^ns^	0.57±(0.30)^ns^	0.49±(0.30)^ns^	0.61±(0.30) ^*^	0.65±(0.34)^ns^
h^2^ b	1.05±(0.37)^*^	0.82±(0.26) ^*^	0.75±(0.26) ^*^	0.68±(0.25) ^*^	0.89±(0.28) ^**^
h^2^ s+d	0.83±(0.12)^**^	0.70±(0.11) ^**^	0.62±(0.11) ^**^	0.65±(0.12) ^**^	0.77±(0.11) ^**^

Va and Vg are the additive and genetic variances that are in liability units (here logit scale) from a generalized linear mixed model.

^a^ variance error for a logit transformation is equal to $\frac{\pi^{2}}{3}$

*: *p*<0.05

**: *p*<0.001

**Table 4:** Narrow and broad genetic correlations for mortality between the testing methods.

	BF	MF	LF	OsHV-1 challenge
BF	_	0.92***	0.92***	0.74***
MF	0.94***	_	0.95***	0.68***
LF	0.93***	0.99***	_	0.72***
OsHV-1 challenge	0.73***	0.69***	0.75***	_

Elements below and above the diagonal are the broad and narrow genetic correlations respectively,

***: *p*<0.001.

## Discussion

According to mortality kinetics, the lack of OsHV-1 DNA detection at deployment, the high amounts of OsHV-1 DNA in moribund oyster tissues at 15 days post-deployment, and the low mortality for the FSF “R” (selected for its higher resistance to OsHV-1 infection), it could be assumed that OsHV-1 was the main factor in the mortality outbreak observed in the field. The same statement is valid for the mortality observed in the OsHV-1 challenge due to the absence of mortality for the oysters injected with seawater.

One of the major concerns for disease resistance in oyster species is that all of the testing methods have failed to provide estimates for heritability for this trait, while numerous studies have clearly shown that resistance was heritable, with a significant gain in survival by breeding the survivors in numerous oysters species: *Haplosporidium nelsoni*, *Perkinsus marinus* and *Roseovarius crassostrea* in *C*. *virginica* [34–36], *Bonamia ostreae* in *Ostrea edulis* [37, 38], and *Bonamia roughleyi* and *Marteilia sydneyi* in *Saccostrea glomerata* [39, 40]. Moderate narrow sense heritabilities were found for mortality in the field (0.49–0.60) in *C*. *gigas* spat (Table 2) and were in agreement with those obtained on the west coast of the USA (h² broad sense at 1 year old = 0.49–0.71) [41], in Japan (h² narrow sense at 1 year old = 0.77) [42], in Australia (h² at 21 months old = 0.68) [43], and, in particular, in France during the Morest program, with a narrow sense heritability for mortality in 6-month-old oysters ranging from 0.47 to 1.08 [22] and a realized heritability ranging from 0.55 to 0.98 [21]. It is also important to note that OsHV-1 has been reported in all of these countries: in the USA [2], in Japan [44], in Australia [4] and in France [45], suggesting that these heritability values for mortality could also be related to the resistance of *C*. *gigas* to OsHV-1 infection. Our study is the first to report genetic variation for OsHV-1 resistance in spat *C*. *gigas* under laboratory conditions, with a moderate narrow heritability (0.61), similar to those obtained in the field (Table 2). It is the first time that heritability for disease resistance in an oyster species has been established. Genetic improvement is then possible in breeding programs to enhance resistance to infection with OsHV-1, a worldwide disease, as this major oyster species is cultivated all around the world. The broad sense heritabilities were larger than the narrow sense heritabilities for all methods, indicating a substantial non-additive genetic variance or variance due to a common environment. However, this could also be attributed to the small number of HSF and FSF within HSF in our study combined with the testing method, as indicated by the large standard error, which decreased for the overall analysis and which were the lowest for the MF and LF methods, as expected. In the future, mixing families at earlier stages, i.e., hatching, could be explored, as demonstrated for growth traits in *C*. *gigas* [46]. Nevertheless, such methods add substantial costs for the mortality trait because they would require pre- and post-planting genotyping of large samples for the estimation of the initial and final family representation due to high variability in family representation produced during spawn, larval and nursery stages [25, 47]. Without these data, it would not be possible to distinguish whether differential mortality among the families is due to the disease or to the hatching/larval/settlement/nursing variability.

For the first time, several methods for testing numerous families were evaluated in a site where OsHV-1 is endemic. Interestingly, high genetic correlations between the three methods suggest no genotype by method interaction in the field (Table 4), indicating that any of the methods could be used to evaluate resistance to viral infection in *C*. *gigas* spat without affecting the ranking of the families. Such a result would be obvious, as no physical separation was possible between the families for all testing methods, indicating a relatively common environment among families and among methods. This is particularly true because massive mortality related to OsHV-1 was mainly observed in spat and juvenile *C*. *gigas* a few weeks post-deployment when the seawater temperature was above 16°C [12, 13]. Nevertheless, our results indicate that it could be possible to significantly reduce the number of bags used in field evaluations from 84 for the BF to only 3 for the LF and MF (Table 1), without affecting the mortality related to OsHV-1 and without observing the amplification effect observed in mixed tanks, as seen in the seabass *Dicentrarchus labrax* [48], common carp *Cyprinus carpio* [24] and rainbow trout *Oncorhynchus mykiss* [49]. The LF method is not worth the effort because it is time consuming to individually label all animals, in comparison to the MF method, for which spat are placed in a soft mesh bag. In the future, estimating oyster resistance to OsHV-1 infection in the field could simply use the MF method, but such a method should not be appropriated for other diseases or other traits.

Field and laboratory trials indicated that most of the families that performed well in the field also performed well in the OsHV-1 challenge in the laboratory. For example, in terms of survival among the 10 best FSF and 10 worst FSF, six and seven were common between the field and laboratory challenge, respectively. This result is in agreement with those obtained in other bivalve species: in *Ostrea edulis* for *Bonamia ostreae* [50], in *C*. *virginica* for *Perkinsus marinus* [51], in *Ruditapes philippinarum* for *Vibrio tapetis* [52] and in *C*. *gigas* for summer mortality in adults [53] and for spat [17]. Meanwhile, our study is the first to validate that the genetic basis of resistance to OsHV-1 infection could be evaluated through a controlled challenge in the laboratory using the injection method, as it mimics the results obtained in classic field evaluations. Such a method could be proposed to improve the selective breeding programs focused on resistance to the viral infection, as has been performed for other important species in aquaculture, such as fish [54–56] and shrimp [57–59]. It is also supposed that the genetic correlation between the field and the laboratory methods could have been higher if both trials had occurred at the same time. Indeed, the field trials occurred in animals at 4.5 months old weighing 2 g, while the OsHV-1 challenge in the laboratory occurred in animals at 7 months old weighing 5 g in average. The mean mortality was 80–82% in the field and 58% in the laboratory. This difference in mortality could be explained by a different intensity of disease exposure (OsHV-1 or other pathogens in the field), different pathways for the disease introduction into the oysters (water-borne contamination versus injection), or higher resistance to OsHV-1 infection in larger and older animals combined with different responses among genotypes [12].

## Supporting Information

S1 Fig Genetic correlation for mortality between methods using the estimate breeding value of each male and each female calculated from BLUP (field methods are BF, MF and LF for bag, mesh and labelled, respectively, and OsHV-1 challenge was conducted under controlled conditions in the laboratory). (EPS)

S1 Table Mortality data of all FSFs recorded at endpoint in each method. (XLSX)

S2 Table Variance components and narrow, broad-sense and combined estimation of heritabilities on the liability scale (h^2^ n, h^2^ b and h^2^ s+d, respectively) (S.E.) using the Dempster & Lerner methods [33] for mortality in *C*. *gigas* spat for each testing method in the field and in the OsHV-1 challenge in the laboratory. (DOCX)

S3 Table Narrow and broad genetic correlations using the BLUP values for each male and for each female. (DOCX)

S1 Text SAS code for the estimation of variance components (Glimmix and Mixed models). (DOCX)
