# Supplementary material for: New Insight for the Genetic Evaluation of Resistance to Ostreid Herpesvirus Infection, a Worldwide Disease, in Crassostrea gigas
Source: PLoS One. 2015 Jun 3;10(6):e0127917. doi: 10.1371/journal.pone.0127917 (PMC4454582; doi:10.1371/journal.pone.0127917)
Supplement: S2 Table — (DOCX) [file pone.0127917.s003.docx]

Table S2: Variance components and narrow, broad-sense and combined estimation of heritabilities on the liability scale (h^2^ n, h^2^ b and h^2^ s+d, respectively) (S.E.) using the Dempster & Lerner methods [33] for mortality in *C. gigas* spat for each testing method in the field and in the OsHV-1 challenge in the laboratory.

| Variance | BF | MF | LF | OsHV-1 challenge | Overall |
| --- | --- | --- | --- | --- | --- |
| Va | 0.06 (±0.05) | 0.05 (±0.03) | 0.04 (±0.03) | 0.11 (±0.06) | 0.08±(0.04) |
| Vg | 0.12 (±0.05) | 0.08 (±0.02) | 0.08 (±0.08) | 0.12 (±0.04) | 0.10±(0.03) |
| Verror | 0.12 | 0.13 | 0.13 | 0.17 | 0.13 |
| Vphenotypic | 0.16 (±0.01) | 0.16 (±0.01) | 0.16 (±0.01) | 0.23 (±0.01) | 0.17±(0.01) |
|  |  |  |  |  |  |
| h^2^ n | 0.39 (±0.37)^ns^ | 0.31 (±0.18)^ns^ | 0.28 (±0.19)^ns^ | 0.72 (±0.36)^ns^ | 0.56±(0.30)^ns^ |
| h^2^ b | 0.86 (±0.32)** | 0.50 (±0.15)** | 0.51 (±0.17)** | 0.76 (±0.28)** | 0.67±(0.22)** |
| h^2^ s+d | 0.62 (±0.11)** | 0.40 (±0.08)** | 0.40 (±0.08)** | 0.74 (±0.14)** | 0.61±(0.10)** |

Va and Vg are the additive and genetic variances on the observed scale estimated from the proc MIXED.

*: *p*<0.05; **: *p*<0.01
