# Supplementary material for: New Insight for the Genetic Evaluation of Resistance to Ostreid Herpesvirus Infection, a Worldwide Disease, in Crassostrea gigas
Source: PLoS One. 2015 Jun 3;10(6):e0127917. doi: 10.1371/journal.pone.0127917 (PMC4454582; doi:10.1371/journal.pone.0127917)
Supplement: S3 Table — (DOCX) [file pone.0127917.s004.docx]

S3 Table: Narrow and broad genetic correlations using the BLUP values for each male and for each female

|  |  |  |  |  |  |  |  |  |  |  |  |  |
| --- | --- | --- | --- | --- | --- | --- | --- | --- | --- | --- | --- | --- |
| Narrow genetic correlations for mortality between testing methods, calculated on BLUP from GLIMMIX procedure (see material and methods). | | | | | | | | | | | | |
| One BLUP have been calculated for each male. | | | | |  |  |  |  |  |  |  |  |
|  |  |  |  |  |  |  |  |  |  |  |  |  |
| Variable | *BF* | *MF* | *LF* | *OsHV1* | *P_BF* | *P_MF* | *P_LF* | *P_OsHV1* | *N_BF* | *N_MF* | *N_LF* | *N_OsHV1* |
| *BF* | 1.00 | 0.88 | 0.92 | 0.77 | _ | <.0001 | <.0001 | <.0001 | 24.00 | 23.00 | 23.00 | 23.00 |
| *MF* | 0.88 | 1.00 | 0.93 | 0.73 | <.0001 | _ | <.0001 | <.0001 | 23.00 | 24.00 | 23.00 | 24.00 |
| *LF* | 0.92 | 0.93 | 1.00 | 0.80 | <.0001 | <.0001 | _ | <.0001 | 23.00 | 23.00 | 23.00 | 23.00 |
| *OsHV1* | 0.77 | 0.73 | 0.80 | 1.00 | <.0001 | <.0001 | <.0001 | _ | 23.00 | 24.00 | 23.00 | 24.00 |
|  |  |  |  |  |  |  |  |  |  |  |  |  |
|  | | | | | | | | | | | | |
|  | | | | | | | | | | | | |
| Broad genetic correlations for mortality between testing methods, calculated on BLUP from GLIMMIX procedure (see material and methods). | | | | | | | | | | | | |
| One BLUP have been calculated for each female. | | | | |  |  |  |  |  |  |  |  |
|  |  |  |  |  |  |  |  |  |  |  |  |  |
| Variable | *BF* | *MF* | *LF* | *OsHV1* | *P_BF* | *P_MF* | *P_LF* | *P_OsHV1* | *N_BF* | *N_MF* | *N_LF* | *N_OsHV1* |
| *BF* | 1.00 | 0.91 | 0.92 | 0.74 | _ | <.0001 | <.0001 | <.0001 | 43.00 | 42.00 | 41.00 | 42.00 |
| *MF* | 0.91 | 1.00 | 0.97 | 0.69 | <.0001 | _ | <.0001 | <.0001 | 42.00 | 46.00 | 41.00 | 44.00 |
| *LF* | 0.92 | 0.97 | 1.00 | 0.72 | <.0001 | <.0001 | _ | <.0001 | 41.00 | 41.00 | 41.00 | 41.00 |
| *OsHV1* | 0.74 | 0.69 | 0.72 | 1.00 | <.0001 | <.0001 | <.0001 | _ | 42.00 | 44.00 | 41.00 | 44.00 |
|  |  |  |  |  |  |  |  |  |  |  |  |  |
