# Supplementary material for: New Insight for the Genetic Evaluation of Resistance to Ostreid Herpesvirus Infection, a Worldwide Disease, in Crassostrea gigas
Source: PLoS One. 2015 Jun 3;10(6):e0127917. doi: 10.1371/journal.pone.0127917 (PMC4454582; doi:10.1371/journal.pone.0127917)
Supplement: S1 Text — (DOCX) [file pone.0127917.s005.docx]

/*********** SAS CODE*******************************************************

This macro have been done to analyze the data from Lionel Dégremont *************************/

/*********** Assitance on this macro should be asked to "jeanbaptistelamy@googlemail.com" ****************/

/*********** Version 12_02_2014 **************************************************************************/

/*AIMS OF THE ANALYSIS*/

/*

Rank each genitors with BLUP (from Generalized linear mixte models),

Estimate Heritability for each experiment,

*/

/*

Definition of Arguments :

max= number of the total files needed to be downloaded

i = macro variable to download all the files

sort = all to merge to files between them. this argument is used in the "MERGE" statement

path_in = files location (usually spatial analysis files)

name_out = the name of the out_files

*/

/*

About the input file :

Input file should be non grouped data (individual phenotype).

Male = numeric columms with the id of each male for each individuals (rows)

Femelle = nuumeric columms with the id of each femelle for each individuals (rows)

famille = idem...

surv = numeric columms with the binary phenotype 1 = vivant and 0 = dead !

*/

/*

proc import out= WORK.biva01

datafile="G:\IFREMER\02-PROJETS\BIVALIFE\INPUT\BIVALIFE_mortality2.xlsx"

dbms=excel REPLACE;

RUN;

data work.biva01;

drop i j k Mortes Vivantes;

do i=1 to 533;

*set bivalife. 'Feuil1$'n;

set work.biva01 ;

if Vivantes^=. then do j=1 to Vivantes;

surv=1;

output;

end;

if Mortes^=. and Mortes > 0 then

do k=1 to Mortes;

surv=0;

output;

end;

keep date_debut date_fin site condition famille male femelle poche surv;

end;

stop;

run;

data biva02 ;

set biva01(where = (famille NE 50)) ;*technical control, this familly have been injected with sea water or OsHV1 ;

run ;

data pocheNG filetNG mixNG cohabNG injNG;

set biva02(keep = site condition famille male femelle poche surv ) ;

if condition = "poche" then output pocheNG ;

if condition ="filet" then output filetNG ;

if condition ="MIX" then output mixNG ;

if condition ="injection" then output injNG ;

run ;

*/

/*

%let data = pocheng ;

%let pathway_out = G:\IFREMER\02-PROJETS\BIVALIFE\OUPTUT\ ;

%let fixed_effect = ;

%let random_effect = male femelle(male) poche(male femelle) ;

%let sort = male femelle poche ;

*/

**%macro** GLIMMIX_BIVALIFE_2 (data =, pathway_out = , fixed_effect = , random_effect = , sort = ) ;

/*0- Create a new directory and set up the environment*/

/*a new create a folder with all the output */

options dlcreatedir;

/*a new create a folder with all the output */

libname newdir "&pathway_out.&data.";

options nonotes nosource nosource2 errors= **10000** ;

ods noresults;

ods listing close;

/*1- analysis */

proc sort data = &data. ;

by &sort. ; run ;

ods pdf file="&pathway_out.\&data.\OUTPUT_&data..pdf";

proc glimmix data=&data. asycov method = LAPLACE ;

class &sort. ;

model surv(event ='1') = &fixed_effect. /s dist = binary link = logit ;

random &random_effect. /s ;

ods output solutionR = BLUP CovParms = cov parameterestimates = BLUES(keep = estimate effect ) fitstatistics = Fit_full asycov = asycov ;

run ;

ods pdf close;

/* merge BLUES and BLUPS estimates */

data blues ;

set blues(where =(effect = "Intercept")) ;

rename estimate = intercept ;

type = **0** ;

drop effect ;

run ;

/* create a dummy variable for the merging step*/

data BLUP ;

set BLUP/*(where =(effect = "male" or effect ="femelle(male)"))*/ ;

type = **0** ;

rename effect = random_effect ;

run ;

data tot ;

merge blup blues;

by type ;

type = _n_ ;

run ;

/*2- BLUP calculation ;

*merge ID and BLUP;

*back transform BLUP in the natural scale [0,1];*/

data tblup ;

set tot ;

*length random_effect $15 ;

BLUP_natural_scale = (exp(estimate))/(**1**+exp(estimate)) ; /** estimate = logit scale;*/

/**more calculation are possible ;*/

drop type DF tvalue probt ;

rename estimate = BLUP_logit_scale ;

run ;

proc sort data= tblup ;

by descending random_effect descending blup_natural_scale ;

run ;

/*trim cov and asycov matrix*/

data cov ;

set cov (where=(CovParm = "male" or CovParm = "femelle(male)" ));

drop StdErr ;

run ;

data asycov ;

set asycov(where=(CovParm = "male" or CovParm = "femelle(male)" ));

keep CovP1 CovP2 ;

run ;

/*3- Heritability estimation ;

*calculate heritability (i will use the notation of Falconer) ;

*from observational components (theta_ ) to causal components (V_);

*theta_sire = 1/4*V_additive ;

*theta_dam(sire) = 1/4V_additive + 1/4 V_dominance + V_maternal_effect ;

*theta_within_familly = 1/2V_additive + 3/4V_dominance + V_within_family_environmental ;

*two equations - Three unknown ;

*Here it is assumed that V_dominance = 0 ;*/

proc iml ;

/**set some vectors ;*/

s_ = {**1** **0** **0**} ; /**design vector to get sire variance ;*/

d_ = {**0** **1** **0**} ; /**design vector to get dam variance ;*/

sd_= {**2** **2** **0**} ; /**design vector to get sire and dam variance ;*/

a_ = {**4** **0** **0**} ;/* *design vector to get additive variance ;*/

g_ = {**0** **4** **0**} ;/* *design vector to get genetic variance ;*/

t_ = {**1** **1** **1** }; /**design vector to get phenotypic variance ;*/

r_ = {**0** **0** **1**} ; /**design vector to get residual variance ;*/

use cov(keep = estimate) ; /**observational variance component;*/

read all var _all_ into a ;

close ;

a = a//((constant('PI')****2**)/**3**) ;/* *add residual variance term (logit scale);*/

use asycov ; /**variance of variance component ;*/

read all var {CovP1 CovP2} into b ;

close ;

/**making an hypothesis about variance of the error variance ;*/

rab = {**0.00001** **0.00001**} ;

b = b//rab ;

rab = {**0.00001** **0.00001** **0.00001**} ;

b = b||t(rab) ;

/***get the total phenotypic variance;*/

V_P = t_*a ; /*add the residual variance (logit scale) ;*/

/**get the variance of V_P and its standard error ;*/

var_V_P = b[+,+] ; /**variance of V_P;*/

SE_V_P = sqrt(var_V_P) ;

/**get the additive variance ;*/

V_A = a_*a ;

/**get the variance of V_A and its standard error ;*/

var_V_A = a_*b*t(a_) ;

SE_V_A = sqrt(var_V_A) ;

/**get the total genetic variance and its standard error ; */

V_G =g_*a ;

/**get the variance of V_G and its standard error ;*/

var_V_G = g_*b*t(g_) ;

SE_V_G = sqrt(var_V_G) ;

/**get the dam and sire variance and its standard error ; */

V_SD =sd_*a ;

/**get the variance of V_SD and its standard error ;*/

var_V_SD = sd_*b*t(sd_) ;

SE_V_SD = sqrt(var_V_SD) ;

/**Sire variances ;*/

V_S = s_*a ;

var_V_S = s_*b*t(s_);

SE_V_S = sqrt(Var_V_S ) ;

/**Dam variances ;*/

V_D = d_*a ;

Var_V_D = d_*b*t(d_) ;

se_V_D = sqrt(Var_V_D) ;

/**get the maternal and/or common environment effect ;*/

V_M = V_D -V_S;

/**design vector for the covariance between Sires and dam component ;*/

cov_VD_VS =s_*b*t(d_) ;

Var_V_M = var_V_S +var_V_D - **2***cov_vd_vs ;

SE_V_M = sqrt(var_V_M);

/**get the micro-environmental effect ;*/

V_E = r_*a - **2***a[**1**,**1**];

/**get a estimation of heritability from paternal component;*/

hp = V_A/V_P ;

/**get a estimation of heritability from maternal component (natural scale);*/

hb = V_G/V_P ;

/**get a estimation of heritability from maternal and paternal component (natural scale);*/

hsd = V_SD/V_P ;

/**PATERNAL ESTIMATION OF HERIDITY get vector of covariance between V_A and V_P;*/

/**get vector of covariance between V_A and V_P;*/

Cov_Var_VA_VP = a_*b*t(t_);

/**standard error of heritability from the delta method ;*/

Var_hp_delta = (hp****2**)*((var_V_A/V_A****2**)+(var_V_P/V_P****2**)-(**2***cov_var_VA_VP/(V_A*V_P)));

if Var_hp_delta < **0** then do ;

Var_hp_delta = **.** ;

SE_hp_delta = **.** ;

end ;

else do ;

SE_hp_delta = sqrt(Var_hp_delta) ;

end ;

/*standard error of heritability from the dickersion formulae ;*/

var_hp_dickerson = var_V_A/(V_P****2**) ;

SE_hp_dickerson =sqrt(var_hp_dickerson);

/**MATERNAL ESTIMATION OF HERIDITY get vector of covariance between V_G and V_P;*/

Cov_Var_VG_VP = g_*b*t(t_);

/**standard error of heritability from the delta method ;*/

Var_hb_delta = (hb****2**)*((var_V_G/V_G****2**)+(var_V_P/V_P****2**)-(**2***cov_var_VG_VP/(V_G*V_P)));

if Var_hb_delta < **0** then do ;

Var_hb_delta = **.** ;

SE_hb_delta = **.** ;

end ;

else do ;

SE_hb_delta = sqrt(Var_hb_delta) ;

end ;

/*standard error of heritability from the dickersion formulae ;*/

var_hb_dickerson = var_V_G/(V_P****2**) ;

SE_hb_dickerson =sqrt(var_hb_dickerson);

/**MATERNAL and PATERNAL ESTIMATION OF HERIDITY get vector of covariance between V_SD and V_P;*/

Cov_Var_VSD_VP = sd_*b*t(t_);

/**standard error of heritability from the delta method ;*/

Var_hsd_delta = (hsd****2**)*((var_V_SD/V_SD****2**)+(var_V_P/V_P****2**)-(**2***cov_var_VSD_VP/(V_SD*V_P)));

if Var_hsd_delta < **0** then do ;

Var_hsd_delta = **.** ;

SE_hsd_delta = **.** ;

end ;

else do ;

SE_hsd_delta = sqrt(Var_hsd_delta) ;

end ;

/*standard error of heritability from the dickersion formulae ;*/

var_hsd_dickerson = var_V_SD/(V_P****2**) ;

SE_hsd_dickerson =sqrt(var_hsd_dickerson);

/*Design is leading by the males are better because

estimates are not contaminated by the maternal effect */

colname = {"sire" "dam" "additive" "genetic" "phenotypic" "maternal" "microenvironmental" "heri_narrow_liability_scale" "heri_broad_liability_scale" "heri_Si_Da_liability_scale" } ;

rowna ={"component" "Variance" "STD_ERR"} ;

sire = V_S//Var_v_s//se_V_s ;

dam = V_D//Var_v_D//se_V_D ;

additive = V_A//Var_v_A//se_V_A ;

genetic = V_G//Var_v_G//se_V_G ;

phenotypic = V_P//Var_v_P//se_V_P ;

maternal = V_M//Var_v_M//se_V_M ;

null ={**.**} ;

microenv = V_E//null//null ;

heri_nar_liability = hp //Var_hp_delta//se_hp_delta ;

heri_bro_liability = hb //Var_hb_delta//se_hb_delta ;

heri_sd_liability = hsd //Var_hsd_delta//se_hsd_delta ;

tot= sire||dam||additive||genetic||phenotypic||maternal||microenv||heri_nar_natural||heri_nar_liability||heri_bro_liability||heri_sd_liability ;

create tot from tot[colname = colname rowname=rowna] ;

append from tot[rowname=rowna] ;

quit ;

/*6- BLUP exportation ; */

/*BLUPS estimation */

proc export data=work.tblup outfile= "&pathway_out.\&data.\BLUP_2.txt" dbms=tab replace ;

run; quit ;

proc export data=work.tot outfile= "&pathway_out.\&data.\heritability_2.txt" dbms=tab replace ;

run; quit ;

options notes source source2 ;

ods listing ;

/*7- PRINT AN ABSTRACT OF THE MACRO RUNNING */

%Put "/*****************************the MACRO analysis_1 is running !************************************/" ;

%Put "See the output dataset in &pathway_out.&data.\ folder " ;

%put "THE MODEL" ;

%Put "proc glimmix data=&data. method = LAPLACE ;

class &sort.; model surv(event ='1') = &fixed_effect. /s dist = binary link = logit ;

random &random_effect. ;

run " ;

%put "BLUPS are in the file &pathway_out.&pathway_out.&data.\BLUP.txt ";

%put "Heritability estimates are in the file &pathway_out.&data.\heritability.txt ";

%Put "/*****************************the MACRO analysis_1 is ending !************************************/" ;

**%mend** ;

/*********** This macro have been done to analyze the data from Lionel Dégremont *************************/

/*********** Assitance on this macro should be asked to "jeanbaptistelamy@googlemail.com" ****************/

/*********** Version 12_02_2014 **************************************************************************/

/*AIMS OF THE ANALYSIS*/

/*

Rank each genitors with BLUP (from Generalized linear mixte models),

Estimate Heritability for each experiments,

*/

/*

Definition of Arguments :

max= number of the total files needed to be downloaded

i = macro variable to download all the files

sort = all to merge to files between them. this argument is used in the "MERGE" statement

path_in = files location (usually spatial analysis files)

name_out = the name of the out_files

*/

/*

About the input file :

Input file should be non grouped data (individual phenotype).

Male = numeric columms with the id of each male for each individuals (rows)

Femelle = nuumeric columms with the id of each femelle for each individuals (rows)

famille = idem...

surv = numeric columms with the binary phenotype 1 = vivant and 0 = dead !

*/

/*

proc import out= WORK.biva01

datafile="G:\IFREMER\02-PROJETS\BIVALIFE\INPUT\BIVALIFE_mortality2.xlsx"

dbms=excel REPLACE;

RUN;

data work.biva01;

drop i j k Mortes Vivantes;

do i=1 to 533;

*set bivalife. 'Feuil1$'n;

set work.biva01 ;

if Vivantes^=. then do j=1 to Vivantes;

surv=1;

output;

end;

if Mortes^=. and Mortes > 0 then

do k=1 to Mortes;

surv=0;

output;

end;

keep date_debut date_fin site condition famille male femelle poche surv;

end;

stop;

run;

data biva02 ;

set biva01(where = (famille NE 50)) ;*technical control, this familly have been injected with sea water ;

run ;

data pocheNG filetNG mixNG cohabNG injNG;

set biva02(keep = site condition famille male femelle poche surv ) ;

if condition = "poche" then output pocheNG ;

if condition ="filet" then output filetNG ;

if condition ="MIX" then output mixNG ;

if condition ="injection" then output injNG ;

run ;

*/

/*

%let data = pocheng ;

%let pathway_out = G:\IFREMER\2-PROJETS\BIVALIFE\OUPTUT\ ;

%let fixed_effect = ;

%let random_effect = male femelle(male) poche(male femelle) ;

%let sort = male femelle ;

*/

**%macro** MIXED_BIVALIFE_2 (data =, pathway_out = ,

fixed_effect = , random_effect = , sort= ) ;

/*0- Create a new directory and set up the environment*/

options dlcreatedir;

libname newdir "&pathway_out.&data.";

options nonotes nosource nosource2 errors= **10000** ;

ods noresults;

ods listing close;

/*1- analysis */

proc sort data = &data. ;

by &sort. ; run ;

/*all analysis in natural (binary) scales*/

ods pdf file="&pathway_out.\&data.\OUTPUT_&data..pdf";

proc mixed data = &data. plots = none asycov covtest ;

class &sort.;

model surv = &fixed_effect. /solution ;

random &random_effect. /solution ;

ods output solutionR = BLUP CovParms = cov solutionF= BLUES(keep = estimate effect ) fitstatistics = Fit_full asycov = asycov ;

run; quit ;

ods pdf close;

/* merge BLUES and BLUPS estimates*/

data blues ;

set blues(where =(effect ="Intercept")) ;

rename estimate = Intercept ;

type = **0** ;

drop effect ;

run ;

/* create a dummy variable for the merging step*/

data BLUP ;

set BLUP ;

type = **0** ;

rename effect = random_effect ;

run ;

data tot ;

merge blup blues;

by type ;

type = _n_ ;

run ;

/*2- BLUP calculation ;

*merge ID and BLUP;

*back transform BLUP in the natural scale [0,1];*/

data tblup ;

set tot ;

/* BLUP are done in natural scale */

/**more calculation are possible ;*/

drop DF tvalue probt ;

rename estimate = BLUP_natural_scale ;

run ;

proc sort data= tblup ;

by descending random_effect descending blup_natural_scale ;

run ;

/*trim cov and asycov matrix*/

data cov ;

set cov (where=(CovParm = "male" or CovParm = "femelle(male)" or CovParm = "Residual"));

drop StdErr ;

run ;

proc transpose data =asycov out = trash(keep =_name_ ) ;

run ;

data _null_ ;

set trash ;

call symput("last",_name_) ;

run ;

%put &last. ;

data asycov ;

set asycov(where=(CovParm = "male" or CovParm = "femelle(male)" or CovParm = "Residual" ));

keep CovP1 CovP2 &last. ;

run ;

/*step calculate the pij...

in our case the sum = the mean */

proc sort data = &data. ;

by femelle male /*famille*/ ;

run ;

proc means data = &data. noprint ;

var surv ;

by femelle male /*famille*/ ;

output out = pij(where =(_stat_ = "MEAN")) ;

run ; quit ;

/*sum the pij across dam within each sire */

proc sort data = pij ;

by femelle male ;

run ;

/*with the freq columns it gives the number of dam */

proc means data = pij(keep =male femelle /*famille*/ surv ) noprint sum ;

var surv ;

by male ;

output out = pij(drop = _type_ ) sum = sum_pij ;

run ; quit ;

data pij ;

set pij(rename = (_freq_ = dam )) ;

D = **1**/dam ;

pro = D*sum_pij ;

run ;

/*3- Heritability estimation ;

*calculate heritability (i will use the notation of Falconer) ;

*from observational components (theta_ ) to causal components (V_);

*theta_sire = 1/4*V_additive ;

*theta_dam(sire) = 1/4V_additive + 1/4 V_dominance + V_maternal_effect ;

*theta_within_familly = 1/2V_additive + 3/4V_dominance + V_within_family_environmental ;

*two equations - Three unknown ;

*Here it is assumed that V_dominance = 0 ;*/

proc iml ;

/**set some vectors ;*/

s_ = {**1** **0** **0**} ; /**design vector to get sire variance ;*/

d_ = {**0** **1** **0**} ; /**design vector to get dam variance ;*/

sd_= {**2** **2** **0**} ; /**design vector to get dam variance ;*/

a_ = {**4** **0** **0**} ;/* *design vector to get additive variance ;*/

g_ = {**0** **4** **0**} ;/* *design vector to get genetic variance ;*/

t_ = {**1** **1** **1** }; /**design vector to get phenotypic variance ;*/

r_ = {**0** **0** **1**} ; /**design vector to get residual variance ;*/

/*utiliser la table cov issu de la proc mixed */

use cov(keep = estimate) ; /**observational variance component;*/

read all var _all_ into a ;

close ;

use asycov ; /**variance of variance component ;*/

read all var _ALL_ into b ;

close ;

/***get the total phenotypic variance;*/

V_P = t_*a ; /*add the residual variance (natural scale) ;*/

/**get the variance of V_P and its standard error ;*/

var_V_P = b[+,+] ; /**variance of V_P;*/

SE_V_P = sqrt(var_V_P) ;

/**get the additive variance ;*/

V_A = a_*a ;

/**get the variance of V_A and its standard error ;*/

var_V_A = a_*b*t(a_) ;

SE_V_A = sqrt(var_V_A) ;

/**get the total genetic variance and its standard error ; */

V_G =g_*a ;

/**get the variance of V_G and its standard error ;*/

var_V_G = g_*b*t(g_) ;

SE_V_G = sqrt(var_V_G) ;

/**get the dam and sire variance and its standard error ; */

V_SD =sd_*a ;

/**get the variance of V_SD and its standard error ;*/

var_V_SD = sd_*b*t(sd_) ;

SE_V_SD = sqrt(var_V_SD) ;

/**Sire variances ;*/

V_S = s_*a ;

var_V_S = s_*b*t(s_);

SE_V_S = sqrt(Var_V_S ) ;

/**Dam variances ;*/

V_D = d_*a ;

Var_V_D = d_*b*t(d_) ;

se_V_D = sqrt(Var_V_D) ;

/**get the maternal and/or common environment effect ;*/

V_M = V_D -V_S;

/**design vector for the covariance between Sires and dam component ;*/

cov_VD_VS =s_*b*t(d_) ;

Var_V_M = var_V_S +var_V_D - **2***cov_vd_vs ;

SE_V_M = sqrt(var_V_M);

/**get the micro-environmental effect ;*/

V_E = r_*a - **2***a[**1**,**1**];

/**get residual variance ;*/

V_R = r_*a ;

/**get a estimation of heritability from paternal component (natural scale);*/

hp = V_A/V_P ;

/**get a estimation of heritability from maternal component (natural scale);*/

hb = V_G/V_P ;

/**get a estimation of heritability from maternal and paternal component (natural scale);*/

hsd = V_SD/V_P ;

/**get a estimation of heritability from paternal component (liability scale). Formulae from Dempster and Lerner;*/

use pij ; /*import the raw data to calculate the p parameter*/

read all var {pro} into mean ;

close ;

p = (sum(mean))/(nrow(mean)) ;

/*Hamaker approximation for the inverse of the CDF*/

c = sqrt( -log(**4***p*(**1**-p)) ) ;

x = (abs(**0.5**-p)*(**1.238***c*(**1**+**0.0262***c)) ) ; /*transformations de x que je ne comprends pas */

z_hamaker = (exp(-**0.5***x****2**))/(sqrt(**2***constant('pi'))) ; /*z = probability of density of a standard normal distribution N(0,1)*/

hpl_dempster = hp*(p*(**1**-p))/(z_hamaker****2**) ; /* underlying scale juste mais ancienne approximation*/

hbl_dempster = hb*(p*(**1**-p))/(z_hamaker****2**) ;

hsdl_dempster = hsd*(p*(**1**-p))/(z_hamaker****2**) ;

/**PATERNAL ESTIMATION OF HERIDITY get vector of covariance between V_A and V_P;*/

/**get vector of covariance between V_A and V_P;*/

Cov_Var_VA_VP = a_*b*t(t_);

/**standard error of heritability from the delta method ;*/

Var_hp_delta = (hp****2**)*((var_V_A/V_A****2**)+(var_V_P/V_P****2**)-(**2***cov_var_VA_VP/(V_A*V_P)));

SE_hp_delta = sqrt(Var_hp_delta) ;

/*standard error of heritability from the dickersion formulae ;*/

var_hp_dickerson = var_V_A/(V_P****2**) ;

SE_hp_dickerson =sqrt(var_hp_dickerson);

/*standard error of heritability with dempster and lerner formulation*/

SE_hpl_dempster = SE_hp_delta*(p*(**1**-p))/(z_hamaker****2**) ;

/**MATERNAL ESTIMATION OF HERIDITY get vector of covariance between V_G and V_P;*/

Cov_Var_VG_VP = g_*b*t(t_);

/**standard error of heritability from the delta method ;*/

Var_hb_delta = (hb****2**)*((var_V_G/V_G****2**)+(var_V_P/V_P****2**)-(**2***cov_var_VG_VP/(V_G*V_P)));

SE_hb_delta = sqrt(Var_hb_delta) ;

/*standard error of heritability from the dickersion formulae ;*/

var_hb_dickerson = var_V_G/(V_P****2**) ;

SE_hb_dickerson =sqrt(var_hb_dickerson);

/*standard error of heritability with dempster and lerner formulation*/

SE_hbl_dempster = SE_hb_delta*(p*(**1**-p))/(z_hamaker****2**) ;

/**MATERNAL and PATERNAL ESTIMATION OF HERIDITY get vector of covariance between V_SD and V_P;*/

Cov_Var_VSD_VP = sd_*b*t(t_);

/**standard error of heritability from the delta method ;*/

Var_hsd_delta = (hsd****2**)*((var_V_SD/V_SD****2**)+(var_V_P/V_P****2**)-(**2***cov_var_VSD_VP/(V_SD*V_P)));

SE_hsd_delta = sqrt(Var_hsd_delta) ;

/*standard error of heritability from the dickersion formulae ;*/

var_hsd_dickerson = var_V_SD/(V_P****2**) ;

SE_hsd_dickerson =sqrt(var_hsd_dickerson);

/*standard error of heritability with dempster and lerner formulation*/

SE_hsdl_dempster = SE_hsd_delta*(p*(**1**-p))/(z_hamaker****2**) ;

/*Design is leading by the males are better because

estimates are not contaminated by the maternal effect */

colname = {"sire" "dam" "additive" "genetic" "phenotypic" "maternal" "microenvironmental" "residual" "heri_narrow_natural_scale" "heri_narrow_liability_scale" "heri_broad_natural_scale" "heri_broad_liability_scale" "heri_Si_Da_natural_scale" "heri_Si_Da_liability_scale" } ;

rowna ={"component" "Variance" "STD_ERR"} ;

sire = V_S//Var_v_s//se_V_s ;

dam = V_D//Var_v_D//se_V_D ;

additive = V_A//Var_v_A//se_V_A ;

genetic = V_G//Var_v_G//se_V_G ;

phenotypic = V_P//Var_v_P//se_V_P ;

maternal = V_M//Var_v_M//se_V_M ;

null ={**.**} ;

microenv = V_E//null//null ;

residual = V_R//null//null ;

heri_nar_natural = hp //Var_hp_delta//se_hp_delta ;

heri_nar_liability = hpl_dempster //null//se_hpl_dempster ;

heri_bro_natural = hb //Var_hb_delta//se_hb_delta ;

heri_bro_liability = hbl_dempster //null//se_hbl_dempster ;

heri_sd_natural = hsd //Var_hsd_delta//se_hsd_delta ;

heri_sd_liability = hsdl_dempster //null//se_hsdl_dempster ;

tot= sire||dam||additive||genetic||phenotypic||maternal||microenv||residual||heri_nar_natural||heri_nar_liability||heri_bro_natural||heri_bro_liability||heri_sd_natural||heri_sd_liability ;

create tot from tot[colname = colname rowname=rowna] ;

append from tot[rowname=rowna] ;

quit ;

/*6- BLUP exportation ; */

/*BLUPS estimation */

proc export data=work.tblup outfile= "&pathway_out.\&data.\BLUP_2.txt" dbms=tab replace ;

run; quit ;

proc export data=work.tot outfile= "&pathway_out.\&data.\heritability_2.txt" dbms=tab replace ;

run; quit ;

options notes source source2 ;

ods listing ;

/*7- PRINT AN ABSTRACT OF THE MACRO RUNNING */

%Put "/*****************************the MACRO analysis_1 is running !************************************/" ;

%Put "See the output dataset in &pathway_out.&data.\ folder " ;

%put "THE MODEL" ;

%Put "proc mixed data = &data. ;

class &sort. ;

model surv = &fixed_effect. ;

random &random_effect. ;

run ; quit ; " ;

%put "BLUPS are in the file &pathway_out.&data.\BLUP.txt ";

%put "Heritability estimates are in the file &pathway_out.&data.\heritability.txt ";

%Put "/*****************************the MACRO analysis_1 is ending !************************************/" ;

**%mend** ;
